# Preclinical Evaluation of Radioiodinated Hoechst 33258 for Early Prediction of Tumor Response to Treatment of Vascular-Disrupting Agents

**DOI:** 10.1155/2018/5237950

**Published:** 2018-02-26

**Authors:** Dongjian Zhang, Meng Gao, Nan Yao, Cuihua Jiang, Wei Liu, Tiannv Li, Shaoli Song, Dejian Huang, Zhiqi Yin, Yunliang Qiu, Qiaomei Jin

**Affiliations:** ^1^Affiliated Hospital of Integrated Traditional Chinese and Western Medicine, Nanjing University of Chinese Medicine, Nanjing 210028, China; ^2^Laboratories of Translational Medicine, Jiangsu Province Academy of Traditional Chinese Medicine, Nanjing 210028, China; ^3^Department of Nuclear Medicine, The First Affiliated Hospital of Nanjing Medical University, Nanjing 210029, China; ^4^Department of Nuclear Medicine, Renji Hospital, Shanghai Jiaotong University School of Medicine, Shanghai 200127, China; ^5^Department of Natural Medicinal Chemistry and Jiangsu Key Laboratory of Natural Medicines, China Pharmaceutical University, Nanjing 210009, China; ^6^Department of Criminal Science and Technology, Nanjing Forest Police College, Nanjing 210023, China

## Abstract

This study aimed to explore the use of ^131^I-Hoechst 33258 (^131^I-H33258) for early prediction of tumor response to vascular-disrupting agents (VDAs) with combretastatin-A4 phosphate (CA4P) as a representative. Necrosis avidity of ^131^I-H33258 was evaluated in mouse models with muscle necrosis and blocking was used to confirm the tracer specificity. Therapy response was evaluated by ^131^I-H33258 SPECT/CT imaging 24 h after CA4P therapy in W256 tumor-bearing rats. Radiotracer uptake in tumors was validated ex vivo using *γ*-counting, autoradiography, and histopathological staining. Results showed that ^131^I-H33258 had predominant necrosis avidity and could specifically bind to necrotic tissue. SPECT/CT imaging demonstrated that an obvious “hot spot” could be observed in the CA4P-treated tumor. Ex vivo *γ*-counting revealed ^131^I-H33258 uptake in tumors was increased 2.8-fold in rats treated with CA4P relative to rats treated with vehicle. Autoradiography and corresponding H&E staining suggested that ^131^I-H33258 was mainly localized in necrotic tumor area and the higher overall uptake in the treated tumors was attributed to the increased necrosis. These results suggest that ^131^I-H33258 can be used to image induction of cell necrosis 24 h after CA4P therapy, which support further molecular design of probes based on scaffold H33258 for monitoring of tumor response to VDAs treatment.

## 1. Introduction

Objective and accurate evaluation of tumor response to anticancer therapy represents one of the biggest challenges in oncology. An early assessment of therapeutic effectiveness will avoid needless exposure of patients to treatment-related side effects and can result in improved patient survival by early intensifying treatment, discontinuing ineffective therapy, or initiating more effective second-line therapy. Furthermore, accurate and early evaluation of tumor response is critical in clinical trials designed to test the effectiveness of new cancer therapies.

Currently in clinical practice, the evaluation of response focuses on volumetric and morphometric changes in the tumor using anatomical imaging according to the Response Evaluation Criteria in Solid Tumors (RECIST) [1]. The major drawback of treatment response evaluation based on RECIST is that it usually takes a few weeks after treatment before tumor shrinkage becomes apparent. As molecular events precede gross morphologic changes, tumor response assessment based on the molecular effects of therapy may show sensitivity and specificity superior to that of anatomic imaging techniques. Fluorine-18 fludeoxyglucose (^18^F-FDG) is the most commonly used PET tracer in clinical practice for monitoring response to therapy in oncology and shows advantages over anatomic imaging in many malignancies [2, 3]. However, ^18^F-FDG is not a cancer-specific probe, and significant uptake can occur in activated inflammatory cells following therapy in both tumor and surrounding tissue [4]. Although several cell death-specific probes have been explored for early evaluation of tumor response to chemotherapy and/or radiotherapy in preclinical and/or clinical studies [5–12], none of them has been studied for early evaluation of tumor response to vascular-disrupting therapies.

In addition to conventional anticancer therapies, the emerging vascular-disrupting therapies are currently receiving considerable attention. Rather than targeting the neoplastic cell population directly, vascular-disrupting agents (VDAs) cause a rapid and selective vascular shutdown in tumors and subsequently induce extensive secondary neoplastic cell death due to ischemia [13, 14]. A lead agent is the combretastatin-A4 phosphate (CA4P), which induces extensive necrosis in a wide variety of preclinical cancer models and significant blood flow reductions in the patient tumors [15, 16]. As secondary tumor necrosis is considered to be indicative of effective treatment for VDAs,* in vivo* noninvasive imaging necrosis using radiotracers can accurately and sensitively predict early responses to VDAs in patients.

**Figure 1:**
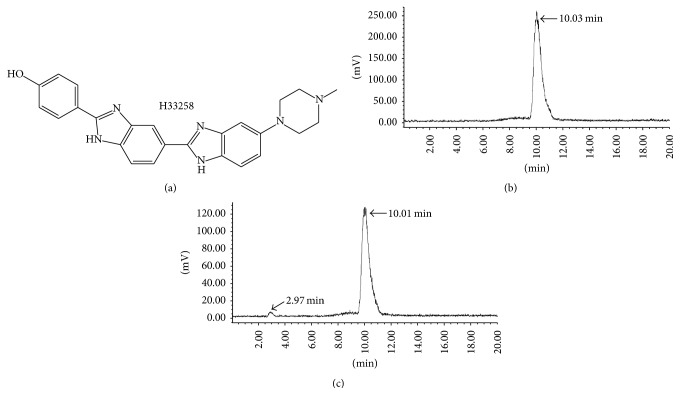
The chemical structure of H33258 (a) and radioactive HPLC profile of ^131^I-H33258 (b) and the corresponding profile of stability study (c). The arrows in (c) denote the peaks corresponding to intact tracer ^131^I-H33258 (10.01 min) and free iodine (2.97 min) after incubation in human serum at 37°C for 24 h.

A common biomarker for necrosis is the intracellular DNA that becomes exposed during necrosis due to disruption of the cell membrane [17, 18]. Hoechst-IR, an imaging agent composed of a biocompatible DNA binding agent Hoechst 33258 (H33258, Figure 1(a)) and a near-infrared dye IR-786, has been demonstrated to allow whole-body fluorescence imaging of necrotic tissue in necrosis-inducing sepsis models by binding exposed DNA [19]. However, for* in vivo* studies, a major weakness with fluorescence imaging is the poor penetration of visible light through skin and tissue. Also, the near-infrared light tends to be scattered as it passes through the sample, leading to the fact that the resolution of whole-body optical images is typically quite poor [20]. In comparison, single photon emission computed tomography (SPECT) imaging can provide much deeper tissue penetration and greatly reduce scattering of the emitted signal and, thus, are more attractive for many types of clinical applications [21]. Therefore, we intend to construct a SPECT imaging probe based on H33258 and explore its potential for early prediction of tumor response to VDAs treatment with CA4P as a representative.

As a preliminary study, iodine-131 was utilized to label H33258 because the labeling method is simple and convenient as well as iodine 131 is easy to obtain for us. And then the necrosis avidity of ^131^I-H33258 was evaluated in mouse models with muscle necrosis. Finally, its potential for early prediction of tumor response to CA4P treatment was explored in W256 tumor-bearing rats.

## 2. Materials and Methods

### 2.1. General

Hoechst 33258 was purchased from Sigma-Aldrich Chemical Co. (St. Louis, MO). Iodogen (1, 3, 4, 6-tetrachloro-3*α*, 6*α*-diphenylglycouril) was obtained from Pierce Biotechnology (ZI Camp Jouven, France). Na^131^I was purchased from HTA Co., Ltd. (Beijing, China). CA4P was purchased from HuaMei Technology Co., Ltd. (Wuhan, China). Radiochemical yield was determined by radio-high performance liquid chromatography (radio-HPLC) with a Waters 2695 pump, a Berthold HERM LB500 radiometric detector, and a GRACE Alltima™ C18 analytical column (250 mm × 4.6 mm, 5 *μ*m) eluted at a flow rate of 1 mL/min under the column temperature of 30°C with the following gradient: an isocratic elution of 10% acetonitrile in water (containing 0.1% TFA) over 5 min, followed by a linear gradient from 10% to 30% acetonitrile in water (containing 0.1% TFA) over 1 min, and then 30% acetonitrile in water (containing 0.1% TFA) over 14 min. For ex vivo studies, radioactivity uptake was measured using the WIZARD^2^ 2470 automated gamma counter (PerkinElmer, Waltham, MA). Kunming mice (25–30 g) and Sprague-Dawley rats (180–200 g) were purchased from Nantong University (Nantong, China) and housed in Experimental Animal Center, Jiangsu Academy of Traditional Chinese Medicine (Nanjing, China). All animal studies were approved by the Institutional Animal Care and Use Committee.

### 2.2. Radiolabeling and* In Vitro* Stability

Labeling was performed using iodogen method. Briefly, H33258 (40 *μ*g) was reconstituted in 40 *μ*L dimethyl sulfoxide and mixed with 40 *μ*L Na^131^I solution (29.6 MBq) in a 50 *μ*g iodogen-coated tube. This reaction mixture was incubated for 10–20 min at room temperature and then diluted with PBS and analyzed by radio-HPLC.

For* in vitro* stability study, 20 *μ*L above reaction solution was mixed with 180 *μ*L human serum and incubated for 24 h at 37°C. Serum proteins were precipitated by adding 400 *μ*L of ethanol and removed by centrifugation (12000 rpm, 10 min). The percentage of intact radiotracer was measured by radio-HPLC.

### 2.3. Animal Models

As a sclerotic agent, ethanol has been used for tumor ablation in human patients and inducing tissue necrosis for experimental studies [22]. Male Kunming mice were intramuscularly injected with 0.2 mL of absolute ethanol in the left hind leg for creating chemically induced muscle necrosis and allowed to recover for 12–15 h after the procedure.

Rat breast carcinoma cell line W256 was obtained from ATCC and maintained in Medium 199 supplemented with 5% horse serum, 100 units/mL penicillin, and 100 *μ*g/mL streptomycin at 37°C in a humidified atmosphere containing 5% CO_2_. W256 cells were injected into abdominal cavity of a SD rat. After incubating for 2 weeks, the animals were executed. Ascites tumor cells were collected and diluted in saline to 5 × 10^6^ cells/mL. W256 cells at a volume of 200 *μ*L were subcutaneously implanted into the right hind flank of SD rats [23]. Tumor size was determined by caliper measurements of long axis (*L*) and short axis (*S*). Tumor volume was calculated following the formula: *V* = (*L* × *S*^2^)/2. When the tumors reached approximately 0.4~0.6 cm^3^, experiments were performed.

### 2.4. Radiotracer Administration

For* in vivo* studies, all animals were given 0.12% potassium iodide in drinking water from 3 days before the experiment until the end of experiment to protect the thyroid gland from taking up free ^131^I. ^131^I-H33258 was formulated by diluting the reaction solution in sterile saline solution. Each mouse model was injected via the tail vein with 0.74 MBq of ^131^I-H33258 for biodistribution studies and each W256 tumor-bearing rat received 7.4 MBq of ^131^I-H33258 for* in vivo* imaging and postmortem verification studies.

### 2.5. Biodistribution Studies in Model Mice

Mice were sacrificed by cervical dislocation in groups (*n* = 4, per group) at 3, 6, 12, and 24 h postinjection (p.i.) of ^131^I-H33258, respectively. Organs of interest were harvested and weighed, and activity uptake of the samples was measured in a gamma counter. The results were expressed as percentage of injected dose per gram of tissue (% ID/g). Blocking studies were also performed in necrotic muscle bearing mice to verify the specificity of ^131^I-H33258 binding. For the blocking studies, mice (*n* = 4) were injected with excess H33258 (10 mg/kg) 45 min prior to injection of 0.74 MBq of ^131^I-H33258 and sacrificed at 3 h p.i. of tracer. Biodistribution studies were then performed as described for other mice groups.

After gamma counting, partially necrotic muscle tissues were cut into sections of 10 *μ*m using a cryostat microtome (Shandon Cryotome FSE; Thermo Fisher Scientific Co., MA) and thaw-mounted on glass slides. These sections were exposed to a high performance phosphor screen (PerkinElmer, Waltham, MA) for 12–24 h. Then the screen was scanned using a Cyclone Plus Phosphor Imager (PerkinElmer, Waltham, MA) and quantification of regions-of-interest on the acquired images was performed using Optiquant™ software (Cyclone; Canberra-Packard, Meriden, CT). After autoradiography, the sections were stained with hematoxylin and eosin (H&E) according to the routine procedure. Sections were then mounted in mounting medium and coverslipped. Digital images of whole sections were taken using a routine digital camera. Photomicrographs were taken with a microscope at ×20 objective (Axioskop; Zeiss, Oberkochen, Germany) (*n* = 4/group).

### 2.6. SPECT/CT Imaging Studies

W256 tumor-bearing rats were given a single intravenous injection of CA4P (20 mg/kg) or vehicle (phosphate buffered saline, PBS) 24 h prior to receiving intravenous injection of ^131^I-H33258. Rats were anaesthetized with intraperitoneal injection of 10% chloral hydrate and secured in prone position near the center of the field of view of the scanner. Static whole-body SPECT images were acquired at 3 h after radiotracer injection using a clinical SPECT/CT scanner (NM/CT670, General Electric Company, Fairfield, CT) equipped with a pinhole high-energy collimator, followed by CT acquisition. The SPECT acquisition parameters are as follows: static image matrix size 128 × 128, energy 140 keV, energy window 10%, and continuous acquisition 48 frames × 11 s/frame. The CT acquisition parameters were 100 keV, 100 mA, and 2.5 mm slice thickness. The SPECT and CT fusion images were obtained and displayed as coronal slices.

### 2.7. Postmortem Analysis

After the scan of SPECT/CT, W256 tumor-bearing rats were immediately sacrificed by intraperitoneal injection of an excess of 10% chloral hydrate. Tumors and other organs of interest were harvested and exsanguinated. Then biodistribution studies were performed as described above. After gamma counting, tumors were cut into sections of 10 *μ*m and then autoradiography and H&E staining studies were carried out as the procedure described above. Digital images of whole sections were taken using a routine digital camera and photomicrographs were taken with a microscope at ×40 objectives (Axioskop; Zeiss, Oberkochen, Germany).

### 2.8. Statistical Analysis

Numerical results were expressed as mean ± standard deviation (SD). Difference between data was compared using Student's *t*-test and *P* < 0.05 was considered statistically significant.

## 3. Results

### 3.1. Radiolabeling and* In Vitro* Stability

H33258 could be labeled with ^131^I, with >97% radiochemical yield and at specific activity of 383.2 MBq/*μ*mol. The radiolabeled tracers were used without further purification for all experiments. For* in vitro* stability assay, ^131^I-H33258 turned out to be stable with a retention time of 10.01 min after incubation in rat plasma at 37°C for 24 h. There is only a small amount of free iodine appearing at 2.97 min (Figure 1).

### 3.2. Biodistribution Studies in Model Mice

Mouse muscular necrosis model had been established to evaluate the necrosis avidity of ^131^I-H33258. The results of the biodistribution studies of ^131^I-H33258 in necrotic muscle bearing mice including corresponding necrotic-to-viable muscle ratios are presented in Table 1. The uptake of ^131^I-H33258 by necrotic muscle was significantly higher than that by viable muscle at each time point (*P* < 0.01) and the peak necrotic-to-viable muscle uptake ratios appeared at 12 p.i. (10.70 ± 1.72). Radioactivity in all other examined organs gradually decreased with the course of the study except for pancreas which demonstrated a slight increase in tracer uptake at 6 h p.i. Despite relatively high initial bladder uptake, radioactivity rapidly decreased from 1.60 ± 0.44% ID/g for 6 h p.i. to 0.09 ± 0.02% ID/g for 12 h p.i.

**Figure 2:**
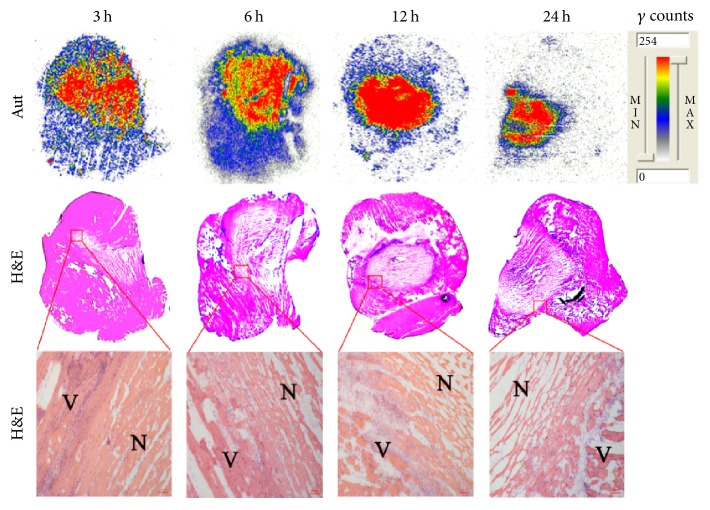
Representative autoradiography (Aut) (upper panels) and corresponding macroscopic (middle panels) and microscopic (lower panels) H&E staining images of 10 *μ*m partially necrotic muscle slices excised from mouse models at 3, 6, 12, and 24 h postinjection of ^131^I-H33258, respectively. N: necrotic muscle; V: viable muscle. Scale bar: 100 *μ*m.

Representative autoradiograms and corresponding H&E staining images of partially necrotic muscle slices are present in Figure 2. The presence of necrosis was confirmed by H&E staining. The contrast between autoradiograph and HE staining showed that ^131^I-H33258 had much higher uptake in necrotic muscle compared with viable muscle at each examined time point, which is consistent with the results of ex vivo biodistribution. These results suggest that ^131^I-H33258 had predominant necrosis avidity and could well identify the necrotic tissue.

### 3.3. Blocking Studies

**Figure 3:**
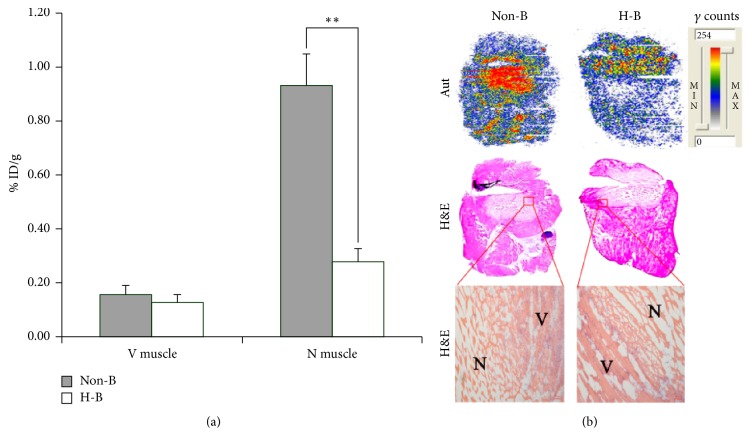
Evaluation of specificity of ^131^I-H33258 binding to necrotic tissue. (a) Uptake of ^131^I-H33258 in nonblocked and 10 mg/kg H33258 blocked viable and necrotic muscle. Data are % ID/g, expressed as mean ± SD (*n* = 4) at 3 h postinjection of ^131^I-H33258. (b) Representative autoradiography (Aut) (upper panels) and corresponding macroscopic (middle panels) and microscopic (lower panels) H&E staining images of 10 *μ*m partially necrotic muscle slices excised from mouse models blocking with or without H33258. Non-B: nonblocked; H-B: H33258 blocked; N: necrotic muscle; V: viable muscle. ^*∗∗*^*P* < 0.01. Scale bar: 100 *μ*m.

To ensure that tracer accumulation in the necrotic tissue was specific, blocking experiments were performed by injecting excess cold H33258 45 min prior to the injection of radiolabeled H33258 in necrotic muscle bearing mice. As shown in Figure 3(a), a decrease of approximate 70% of radioactivity in the necrotic muscle was observed in the presence of excess H33258 compared with the unblocked group, confirming the specific binding of ^131^I-H33258. This result was further confirmed by autoradiography and corresponding H&E staining in Figure 3(b). The semiquantitative analysis of autoradiographs showed that the necrotic-to-viable muscle uptake ratios of H33258-blocking and unblocked group were 2.2 and 5.8, respectively. The above results suggest that ^131^I-H33258 and unlabeled H33258 may have the same binding target in necrotic tissues.

### 3.4. SPECT/CT Imaging

**Figure 4:**
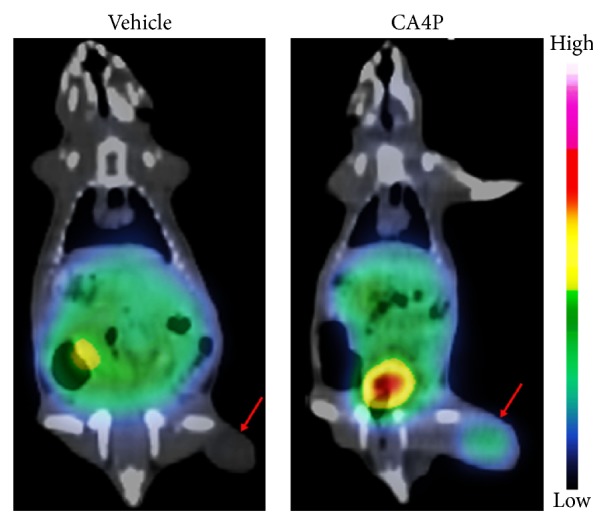
Representative coronal SPECT/CT images of W256 tumor-bearing rats 3 h following injection of ^131^I-H33258. Tumors were treated with vehicle or CA4P 24 h prior to injection of ^131^I-H33258 and obvious tracer uptake was observed in CA4P treated tumor on right hind flank. Tumors are denoted by red arrows.

SPECT/CT imaging was conducted to examine the potential of ^131^I-H33258 for* in vivo* evaluation of treatment response in tumors. Representative coronal SPECT/CT images of W256 tumor-bearing rats treated with vehicle or CA4P at 3 h p.i. of the tracer are shown in Figure 4. Hotspots were clearly visible in the treated tumor with increased necrosis as evidenced by postmortem H&E staining, while no obvious uptake was observed in the vehicle-treated tumor with only a small amount of spontaneous necrosis, suggesting that ^131^I-H33258 could specifically image tumor necrosis induced by CA4P treatment.

### 3.5. Postmortem Analysis

**Figure 5:**
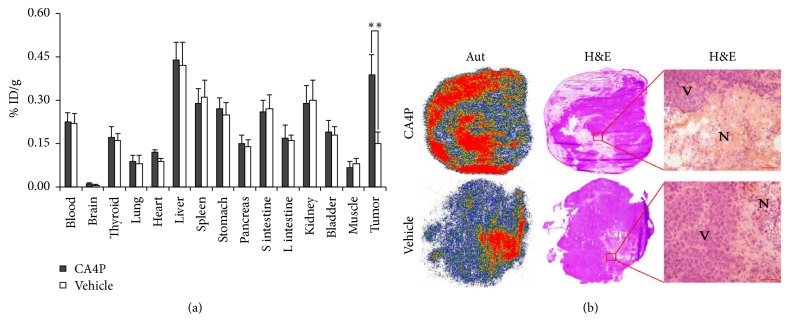
(a) Ex vivo biodistribution analysis of ^131^I-H33258 in W256 tumor-bearing rats after SPECT/CT imaging. Data are % ID/g, expressed as mean ± SD (*n* = 4). S intestine: small intestine; L intestine: large intestine. (b) Representative autoradiography (Aut) (left columns) and corresponding macroscopic (middle columns) and microscopic (right columns) H&E staining images of 10 *μ*m tumor slices excised from W256 tumor-bearing rats treated with vehicle or CA4P 24 h prior to administration of ^131^I-H33258. N: necrotic tumor; V: viable tumor. ^*∗∗*^*P* < 0.01, tumor uptake of ^131^I-H33258 in CA4P treatment group compared with control group. Scale bar: 50 *μ*m.

Ex vivo biodistribution results after SPECT/CT imaging are presented in Figure 5(a). Compared with control group, CA4P treatment resulted in a significant higher tumor uptake of ^131^I-H33258 (*P* < 0.01). The tumor-to-blood and tumor-to muscle uptake ratios were 1.7 and 5.7, respectively, for CA4P treatment group and 0.7 and 1.9, respectively, for control group. There was no statistical difference in other all examined organs/tissues uptake between control group and CA4P treatment group (*P* > 0.05).

Autoradiograms and corresponding H&E staining images of tumor slices from W256 tumor-bearing rats treated with vehicle or CA4P are shown in Figure 5(b). Tumors of rats treated with CA4P showed increased levels of necrosis in comparison with tumors of vehicle-treated rats as evidenced by H&E staining. The contrast between autoradiographs and H&E staining images showed that the intense signal of autoradiography was mainly localized in necrotic tumor area and the necrotic-to-viable tumor signal ratios were 3.8 and 3.9 for treatment group and control group, respectively, which suggested that ^131^I-H33258 could specifically delineate tumor necrosis and the higher overall uptake in the treated tumors was attributed to the increased necrosis.

## 4. Discussion

Vascular-disrupting agents (VDAs) are a class of molecules, which can selectively cause tumor vascular shutdown and subsequently trigger a cascade of tumor cell death in a broad range of experimental tumors [14]. Initial efficacy and toxicity may be tested* in vitro*, but a crucial step is evaluation in animal models prior to translation to human subjects. Noninvasive monitoring of treatment response, including insight into typical intratumoral changes after the use of VDAs, is of major practical importance.

A number of efforts have been made to noninvasively monitor tumor response to VDAs. For instance, dynamic contrast-enhanced MRI has been widely applied in the development of the VDAs [24]. However, it is mainly based on changes in tissue water signal in response to distribution of paramagnetic contrast agent. Therefore, it directly reflects the change of tumor perfusion rather than the death of tumor cells. Moreover, diffusion-weighted MR imaging has been explored for noninvasive evaluation of vascular-disrupting treatment and demonstrated to be able to discriminate between nonperfused but viable and necrotic tumor tissues [25, 26], which provide a powerful measure for assessment of VDAs treatments. More recently, dynamic bioluminescent imaging (BLI) has been introduced to investigate the acute effects of vascular-disrupting agents. Consistent results were found by Zhao et al. using dynamic BLI and dynamic contrast-enhanced MRI following administration of CA4P [27]. BLI of tumors was consistent with vascular shutdown after 2 h following administration of CA4P while changes in fluorescence imaging were considerably delayed consistent with cell death, as opposed to vascular shutdown [28], which suggested that BLI directly reflected vascular extent and flow in tumors rather than the death of tumor cells. Besides, the primary drawback of this approach is the need for luciferase expressing cells.

*In vivo* noninvasive imaging tumor necrosis, an indication of effective treatment, may accurately and early evaluate tumor responses to VDAs. Exposed DNA is a common biomarker for necrosis with considerable potential for diagnosis and therapy of necrosis-related diseases [19, 29–32]. In a previous study reported by Dasari et al., Hoechst-IR, composed of a biocompatible DNA binding agent H33258 and a near-infrared dye IR-786, allowed whole-body fluorescence imaging of necrotic tissue when evaluated in necrosis-inducing sepsis* in vivo* models [19]. In the following studies, H-Gemcitabine and H-IGF1 had been designed for targeting gemcitabine and IGF-1 to the necrotic core of tumors and the infarcted heart, respectively, via the Hoechst moiety [30, 31]. The favorable results of utilizing H33258 as a targeting vector motivated us to explore its potential for early evaluation of tumor response to VDAs treatment by constructing a SPECT/CT tracer.

Although numerous radionuclides are available for radiolabeling, as a preliminary exploration, we chose radioactive iodine to label H33258 because the labeling can be easily achieved by one-step reaction using iodogen which served as an oxidizing agent due to the presence of phenol ring in H33258. Iodine-131 instead of iodine-123, which is more suitable for SPECT/CT imaging, was used because of the relative ease of acquirement and its much longer half-life (*t*_1/2_ = 8.0 days), which could make experiment significantly easier by preventing decay-induced loss.

In the present study, ^131^I-H33258 was shown to have prominent targeting for necrosis in* in vivo* mouse models with muscle necrosis. Furthermore, statistically significant decreased uptake of ^131^I-H33258 in necrotic muscle was observed in the presence of excess cold H33258 compared with the control, which was consistent with our previous research results on other necrosis avid probes [33, 34] and suggested that ^131^I-H33258 and cold H33258 might share the same specific target in necrotic tissues. In the previous studies, H33258 had been used for delivering imaging group or therapeutic drugs to necrotic tissues by binding to extracellular DNA [19, 30–32]. These led us to think that the necrosis targeting of ^131^I-H33258 might be due to its binding to exposed DNA present in the necrotic tissues.

In order to explore the potential of ^131^I-H33258 for early evaluation of tumor response to VDAs treatment, SPECT/CT imaging was performed in W256 tumor-bearing rats treated with CA4P. We found marked increases in ^131^I-H33258 uptake in tumors during the 24 h after CA4P treatment, which accurately reflected induction of cell necrosis as validated by ex vivo histology (Figure 5(b)). The good correlation between ^131^I-H33258 tumor uptake and histologic proof of necrotic cell death demonstrated that ^131^I-H33258 could serve as a new radiotracer for evaluating tumor response to VDAs treatment* in vivo*.

Furthermore, we found that the increase in ^131^I-H33258 accumulation in treated tumors versus the vehicle group was in the range of other cell death-imaging radiotracers (1.3- to 2.8-fold) [5–12]. For example, in a systematic study comparing different cell death-imaging agents, Whitney et al. found a 1.4- to 2.1-fold increase in ^99m^Tc-annexin V and ^18^F-C-SNAT uptake in lymphoma tumors of mice treated with etoposide [8]. Although ^99m^Tc-annexin V proceeded to clinical trials, the inadequate biodistribution profile and low target-to-background ratio [35] resulting from the large protein structure of annexin V (36 kDa) led to the failure of ^99m^Tc-annexin V to reach clinical practice. Another example was the radioiodinated histone H1-binding hexapeptide named [^124^I]ApoPep-1, which demonstrated a 1.6- and 2.3-fold uptake increase in A549 tumors [12] and H460 tumors [9] of mice treated with doxorubicin, respectively. However, despite the considerable potential of [^124^I]ApoPep-1 as a cell death-imaging probe, its low-affinity for the target limits further translation to the clinic [36]. Our present study indicated that radiolabeled H33258 could be utilized as a new radiotracer for* in vivo* necrotic cell death imaging.

As a diagnostic radionuclide, its nuclear physical properties should be superior. As we know, iodine-131 is a beta decay nuclide and mainly used for the purpose of therapy or integration of diagnosis and therapy. The high-energy gamma ray (364 keV) leads to the fact that the quality of imaging is suboptimal and the beta particles emitted with 99% abundance are detrimental to normal tissue* in vivo*. Among the various radioactive isotopes of iodine, iodine-123 with a physical half-life of 13.2 h, the main photon energy of 159 keV and without beta emission, is best suited for the preparation of imaging probes for the diagnosis of nuclear medicine. Therefore, iodine-123 or other gamma ray emitting radionuclides with shorter physical half-life such as ^99m^Tc can be used for constructing imaging probes that are more appropriate for diagnostic purpose in the future research.

## 5. Conclusion

Our present results suggest that ^131^I-H33258 has predominant necrosis avidity and can visualize induction of cell necrosis by SPECT/CT imaging 24 h after CA4P therapy, which support further molecular design of probes based on scaffold H33258 for monitoring of tumor response to VDAs treatment.

## Figures and Tables

**Table 1:** Biodistribution of ^131^I-H33258 in necrotic-muscle bearing mice models.

Organ	Uptake (% ID/g)			
	3 h	6 h	12 h	24 h
Blood	0.77 ± 0.13	0.46 ± 0.01	0.13 ± 0.04	0.06 ± 0.02
Brain	0.03 ± 0.01	0.02 ± 0.02	0.01 ± 0.00	0.01 ± 0.00
Thyroid	0.57 ± 0.11	0.34 ± 0.08	0.23 ± 0.03	0.05 ± 0.01
Lung	0.48 ± 0.10	0.39 ± 0.09	0.32 ± 0.01	0.11 ± 0.02
Heart	0.18 ± 0.06	0.12 ± 0.01	0.06 ± 0.01	0.04 ± 0.01
Liver	1.49 ± 0.15	1.02 ± 0.30	0.57 ± 0.10	0.24 ± 0.04
Spleen	1.14 ± 0.15	0.67 ± 0.11	0.34 ± 0.37	0.14 ± 0.03
Stomach	1.62 ± 0.12	0.81 ± 0.17	0.44 ± 0.01	0.17 ± 0.08
Pancreas	0.30 ± 0.06	0.32 ± 0.06	0.07 ± 0.00	0.05 ± 0.01
S intestine	0.61 ± 0.14	0.40 ± 0.11	0.06 ± 0.01	0.04 ± 0.02
L intestine	0.47 ± 0.10	0.24 ± 0.20	0.06 ± 0.02	0.04 ± 0.02
Kidney	1.11 ± 0.16	0.84 ± 0.13	0.56 ± 0.05	0.17 ± 0.05
Bladder	2.13 ± 0.28	1.60 ± 0.44	0.09 ± 0.02	0.05 ± 0.04
V muscle	0.16 ± 0.03	0.10 ±0.03	0.03 ± 0.01	0.02 ± 0.01
N muscle	0.93 ± 0.12^*∗∗*^	0.61 ± 0.08^*∗∗*^	0.35 ± 0.03^*∗∗*^	0.24 ± 0.04^*∗∗*^
N/V ratio	5.81 ± 0.55	6.19 ± 0.92	10.70 ± 1.72	9.75 ± 1.10

Data are presented as mean ± SD for four mice. S intestine: small intestine; L intestine: large intestine; V muscle: viable muscle; N muscle: necrotic muscle; N/V ratio: necrotic/viable muscle ratio. ^*∗∗*^*P* < 0.01, compared with the uptake values of ^131^I-H33258 in viable muscle at each time point.
